# Not all 1p/19q non-codeleted oligodendroglial tumors are astrocytic

**DOI:** 10.18632/oncotarget.11378

**Published:** 2016-08-18

**Authors:** Yan-Xi Li, Zhifeng Shi, Abudumijiti Aibaidula, Hong Chen, Qisheng Tang, Kay Ka-Wai Li, Nellie Yuk-Fei Chung, Danny Tat-Ming Chan, Wai Sang Poon, Ying Mao, Jinsong Wu, Liangfu Zhou, Aden Ka-yin Chan, Ho-Keung Ng

**Affiliations:** ^1^ Department of Anatomical and Cellular Pathology, Chinese University of Hong Kong, Hong Kong, China; ^2^ Shenzhen Research Institute, Chinese University of Hong Kong, Hong Kong, China; ^3^ Neurosurgery Division, Department of Surgery, Chinese University of Hong Kong, Hong Kong, China; ^4^ Department of Neurosurgery, Huashan Hospital, Fudan University, Shanghai, China; ^5^ Department of Neuropathology, Huashan Hospital, Fudan University, Shanghai, China

**Keywords:** oligodendroglial tumors, intact 1p/19q, TERT, IDH, Pathology Section

## Abstract

Although 1p/19q codeletion is the genetic hallmark defining oligodendrogliomas, approximately 30-40% of oligodendroglial tumors have intact 1p/19q in the literature and they demonstrate a worse prognosis. This group of 1p/19q intact oligodendroglial tumors is frequently suggested to be astrocytic in nature with TP53 and ATRX mutations but actually remains under-investigated. In the present study, we provided evidence that not all 1p/19q intact oligodendroglial tumors are astrocytic through histologic and molecular approaches. We examined 1p/19q status by FISH in a large cohort of 337 oligodendroglial tumors and identified 39.8% lacking 1p/19q codeletion which was independently associated with poor prognosis. Among this 1p/19q intact oligodendroglial tumor cohort, 58 cases demonstrated classic oligodendroglial histology which showed older patient age, better prognosis, association with grade III histology, PDGFRA expression, TERTp mutation, as well as frequent IDH mutation. More than half of the 1p/19q intact oligodendroglial tumors showed lack of astrocytic defining markers, p53 expression and ATRX loss. TP53 mutational analysis was additionally conducted in 45 cases of the 1p/19q intact oligodendroglial tumors. Wild-type TP53 was detected in 71.1% of cases which was associated with classic oligodendroglial histology. Importantly, IDH and TERTp co-occurred in 75% of 1p/19q intact, TP53 wild-type oligodendrogliomas, highlighting the potential of the co-mutations in assisting diagnosis of oligodendrogliomas in tumors with clear cell morphology and non-codeleted 1p/19q status. In summary, our study demonstrated that not all 1p/19q intact oligodendroglial tumors are astrocytic and co-evaluation of IDH and TERTp mutation could potentially serve as an adjunct for diagnosing 1p/19q intact oligodendrogliomas.

## INTRODUCTION

Oligodendroglial tumors comprise of oligodendrogliomas and oligoastrocytomas and are associated with favorable clinical behaviors compared with astrocytomas and some can be chemosensitive [1]. Codeletion of 1p and 19q is a characteristic genetic signature which is observed in 39-70% of oligodendrogliomas and 21-59% of oligoastrocytomas in most series [2-4]. Codeletion is associated with longer survival and is also a predictive marker for response to chemotherapy with procarbazine, lomustine and vincristine [1, 5].

Aside from codeletion of 1p/19q, oligodendroglial tumors are strongly associated with mutations of *IDH1/2*, *TERT* promoter (*TERTp*) and they are also associated with methylation of *MGMT* and upregulation of PDGFRA [6-8]. *TERTp* mutations are regarded as a common mechanism of upregulation of telomerase in primary glioblastoma and oligodendroglial tumors [9]. 1p/19q codeleted and *TERTp* mutated tumors are mutually exclusive with tumors exhibiting mutations in *ATRX* (alpha thalassemia mental retardation syndrome X linked) and *TP53*. The latter two are usually regarded as markers of astrocytic lineage [7, 10-12].

The fact remains that in most series with many coming from important international trials, 30-40% of oligodendroglial tumors are 1p/19q non-codeleted and these tumors exhibit a worse prognosis [1, 13-15]. It has been proposed that most, if not all, of these non-codeleted tumors are actually astrocytic in nature with either *TP53* or *ATRX* mutations [11]. However, there have been few large-scale studies on molecular characterization of oligodendroglial tumors without 1p/19q codeletion. They make up a significant proportion of “oligodendroglial tumors” diagnosed in international series and daily practice and there is no standard-of-care treatment strategy. In this study, we aim to characterize the molecular and clinical features of this neglected group, interrogating them with standard biomarkers and correlating with overall survival.

## RESULTS

### Cohort characteristics

The 337 cases of oligodendroglial tumors included 125 oligodendrogliomas (WHO grade II), 105 oligoastrocytomas (WHO grade II), 72 anaplastic oligodendrogliomas (WHO grade III) and 33 anaplastic oligoastrocytomas (WHO grade III). The mean and median ages of the cohort were 43.1 and 43.0 years, respectively (range 5 to 70 years). The male to female ratio was 1:0.77. Operation data was available in 80.9% (271/335) of patients, with 72.7% (197/271) of patients received total resection and 27.3% (74/271) of patients received non-total resection. Adjuvant treatment data was available in 75.8% (254/335) of patients, with 76% (193/254) of patients receiving radiotherapy and 57.1% (145/254) of patients receiving chemotherapy. Survival data was available in 74.3% (249/335) of patients, with median follow-up and median overall survival being 8.2 years and 10.6 years, respectively.

### Clinical and molecular differences between 1p/19q codeleted and non-codeleted oligodendroglial tumors

**Figure 1:**
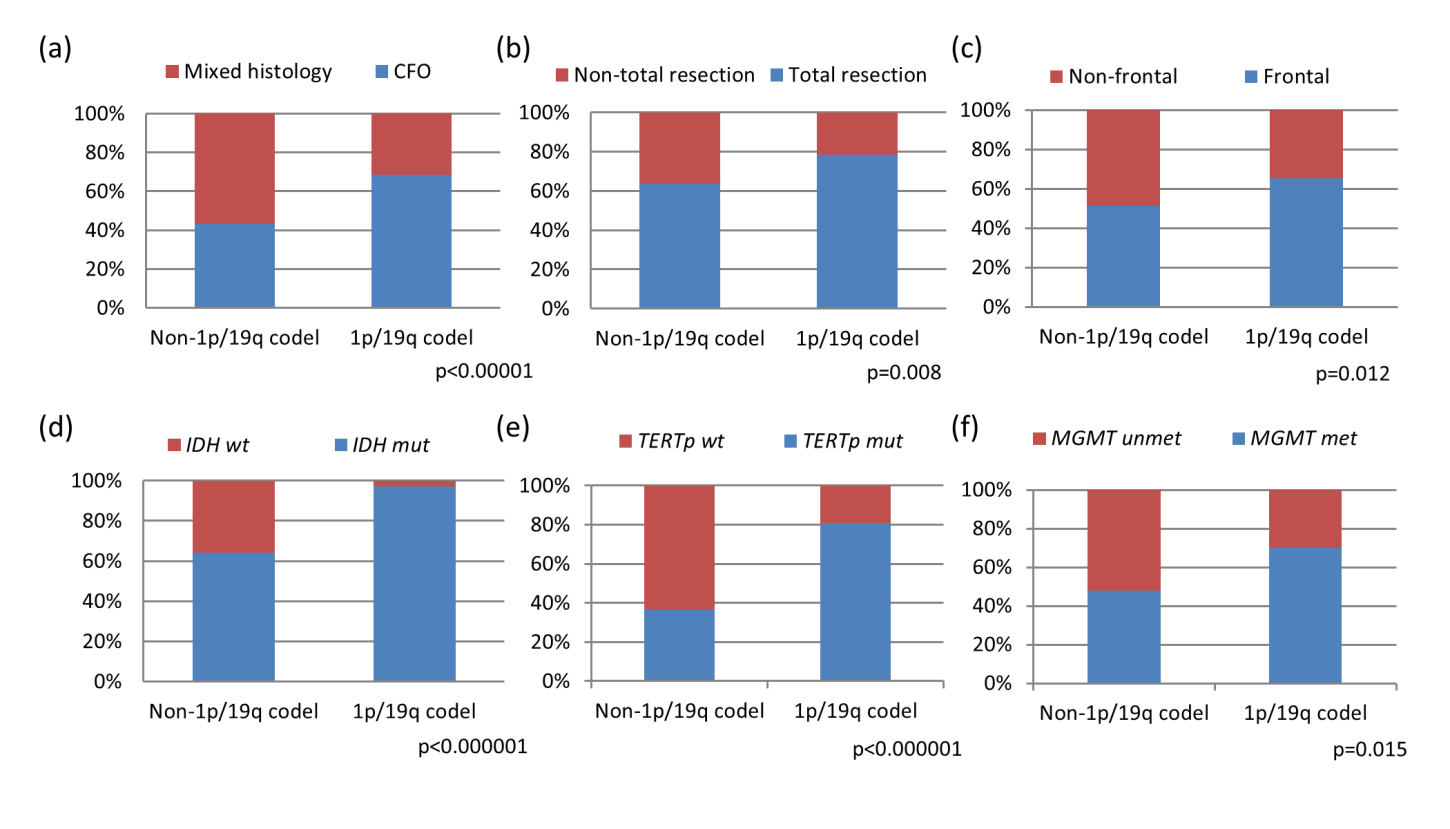
Clinical and molecular characteristics of oligodendroglial tumors based on 1p/19q codeletion status Tumors with 1p/19q codeletion showed significant associations with classical oligodendroglial histology (*p* < 0.00001) **a.** frontal lobe localization (*p* = 0.012) **c.** and were more amenable to total surgical resection (*p* = 0.008) **b.** 1p/19q codeleted tumors also exhibited co-occurring associations with *IDH* mutation (*p* < 0.000001) **d.** *TERTp* mutation (*p* < 0.000001) **e.** and *MGMT* promoter methylation (*p* = 0.015) f. CFO, classic for oligodendroglial morphology; mixed, mixed oligoastrocytic histology; mut, mutant; wt, wild-type; met, methylated; unmet, unmethylated.

Chromosomal 1p and 19q status were examined in all 337 samples of oligodendroglial tumors, with 1p loss detected in 63.8% (215/337) and 19q loss detected in 62.0% (209/337) of the cohort. Combined 1p/19q codeletion was observed in 60.2% (203/337) of cases including 95 oligodendrogliomas, 44 anaplastic oligodendrogliomas, 51 oligoastrocytomas and 13 anaplastic oligoastrocytomas. 1p loss only and 19q loss only were found in 8.2% and 4.5% among the 134 1p/19q non-codeleted oligodendroglial tumors, respectively. Clinical and molecular characteristics were summarized according to 1p/19q codeletion status in Table 1. Comparing the variables between 1p/19q codeleted and non-codeleted oligodendroglial tumors, tumors with codeletion showed significant associations with classic oligodendroglial histology (*p* < 0.00001), frontal lobe localization (*p* = 0.012), and were more amenable to total surgical resection (*p* = 0.008). Molecularly, 1p/19q codeleted tumors also exhibited co-occurring associations with *IDH* mutation (*p* < 0.000001), *TERTp* mutation (*p* < 0.000001), and *MGMT* promoter methylation (*p* = 0.015) (Figure 1a to 1f).

**Table 1:** Clinical and molecular characteristics of oligodendroglial tumors according to 1p/19q status

		**n**	**1p/19q non-codeleted OTs**	**1p/19q codeleted OTs**	**All OTs**
Gender (male / female)		337	74 / 60	115 / 88	189 / 148
Age (mean / median / range)		337	40.8 / 42 / 5 - 72 years	44.6 / 44 / 21 - 75 years	43.1 /43 / 5 - 75 years
Histologic type		337			
	Oligodendroglioma		58 (43.3%)	139 (68.5%)	197 (58.5%)
	Oligoastrocytoma		76 (56.7%)	64 (31.5%)	140 (41.5%)
Histologic grade		337			
	II		86 (64.2%)	146 (71.9%)	232 (68.8%)
	III		48 (35.8%)	57 (28.1%)	105 (31.2%)
Tumor location		334			
	Frontal		67 (50.8%)	126 (62.4%)	193 (57.8%)
	Temporal		20 (15.2%)	23 (11.4%)	43 (12.9%)
	Parietal		5 (3.8%)	11 (5.4%)	16 (4.8%)
	Occipital		5 (3.8%)	3 (1.5%)	8 (2.4%)
	More than one cerebral lobe		21 (15.9%)	27 (13.4%)	48 (14.4%)
	Other locations		14 (10.6%)	12 (5.9%)	26 (7.8%)
Operation		273			
	Total resection		74 (63.8%)	123 (78.3%)	197 (72.2%)
	Non-total resection		42 (36.2%)	34 (21.7%)	76 (27.8%)
Adjuvant therapy		256			
	Concomitant chemo-radiotherapy		47 (45.2%)	84 (55.3%)	131 (51.2%)
	Radiotherapy only		23 (22.1%)	41 (27.0%)	64 (25.0%)
	Chemotherapy only		4 (3.8%)	9 (5.9%)	13 (5.1%)
	No adjuvant therapy		30 (28.8%)	18 (11.8%)	48 (18.8%)
*IDH*		305			
	*IDH1* mut		70 (58.3%)	172 (93.0%)	242 (79.3%)
	*IDH2* mut		7 (5.8%)	8 (4.3%)	15 (4.9%)
	wt		43 (35.8%)	5 (2.7%)	48 (15.7%)
*TERT*p		248			
	C228T		21 (23.3%)	91 (57.6%)	112 (45.2%)
	C250T		12 (13.3%)	37 (23.4%)	49 (19.8%)
	wt		57 (63.3%)	30 (19.0%)	87 (35.1%)
mut, mutant; wt, wild-type; n, number of cases with data available.					

Univariate survival analysis was conducted in the cohort according to the clinical and molecular variables (Table 2). Oligodendroglial tumors with 1p/19q codeletion exhibited significantly longer overall survival than 1p/19q non-codeleted tumors (median OS 11.8 years *vs* 7.0 years, *p* < 0.00001). Other favorable prognostic factors in univariate analysis included age ≤ 50 years (*p* < 0.000001), histologic grade II (*p* > 0.000001), and *IDH* mutation (*p* = 0.001). Trend of better prognosis was observed in tumors with classic oligodendroglial histology (*p* = 0.059) and *TERTp* mutation (*p* = 0.053). Independent favorable prognostic value of 1p/19q codeletion was demonstrated in multivariate analysis by adjusting with significant factors in univariate analysis (Table 3). Favorable prognostic value of 1p/19q codeletion (HR = 0.51, *p* = 0.015) was independent of age (*p* < 0.000001), histologic grade (*p* < 0.00001), histologic type (*p* = 0.003), *IDH* status (*p* = 0.014), and *TERTp* status (*p* = 0.415).

**Table 2:** Univariate analysis of clinical and molecular variables in oligodendroglial tumors

		**n**	**HR**	**[95%CI]**	**Median OS (years)**	*****p*****
Gender	Male	137	1		10.1	0.18
	Female	106	0.755	[0.499 - 1.141]	11.8	
						
Age	≤ 50 years	181	1		11.8	<0.000001
	> 50 years	62	3.289	[2.173 - 4.98]	5.9	
						
Histologic grade	Grade II	169	1		11.8	<0.000001
	Grade III	74	3.84	[2.517-5.859]	6.7	
						
Histologic type	Oligodendroglial	132	1		11	0.059
	Mixed oligoastrocytic	111	1.481	[0.982-2.234]	10	
						
Frontal involvement	Yes	173	1		10.6	0.238
	No	69	1.302	[0.839 - 2.02]	7.8	
						
Operation	Total resection	173	1		11.3	0.172
	Non-total resection	67	1.345	[0.878 - 2.059]	9.7	
						
Adjuvant therapy	Concomitant chemo-radiotherapy	127	1		10.6	0.361
	Radiotherapy only	57	1.512	[0.867 - 2.639]	8.8	
	Chemotherapy only	9	1.464	[0.869 - 2.464]	NR	
	No adjuvant therapy	38	0.872	[0.21 - 3.63]	10.1	
						
1p/19q	codeleted	143	1		11.8	<0.00001
	non-codeleted	100	2.563	[1.689 - 3.889]	7	
						
*IDH*1/2	mut	195	1		10.6	0.001
	wt	25	2.553	[1.454 - 4.483]	3.8	
						
*TERT*p	mut	121	1		11.1	0.053
	wt	58	1.638	[0.988 - 2.715]	10.1	
mut, mutant; wt, wild-type; n, number of cases with data available.						

**Table 3:** Multivariate analysis of clinical and molecular variables in oligodendroglial tumors

		**HR**	**[95%CI]**	***p***
Age		1.074	[1.046-1.103]	<0.000001
				
Histologic grade	Grade II	1		<0.00001
	Grade III	3.736	[2.087-6.688]	
				
Histologic type	Oligodendroglial	1		0.003
	Mixed oligoastrocytic	2.413	[1.359-4.284]	
				
1p/19q	codeleted	0.51	[0.296-0.879]	0.015
	non-codeleted	1		
				
*IDH*1/2	mut	0.364	[0.162-0.817]	0.014
	wt	1		
				
*TERT*p	mut	0.786	[0.441-1.402]	0.415
	wt	1		
mut, mutant; wt, wild-type; n, number of cases with data available.				

### Presence of classic oligodendroglial morphology in 1p/19q non-codeleted gliomas with distinct features

**Figure 2:**
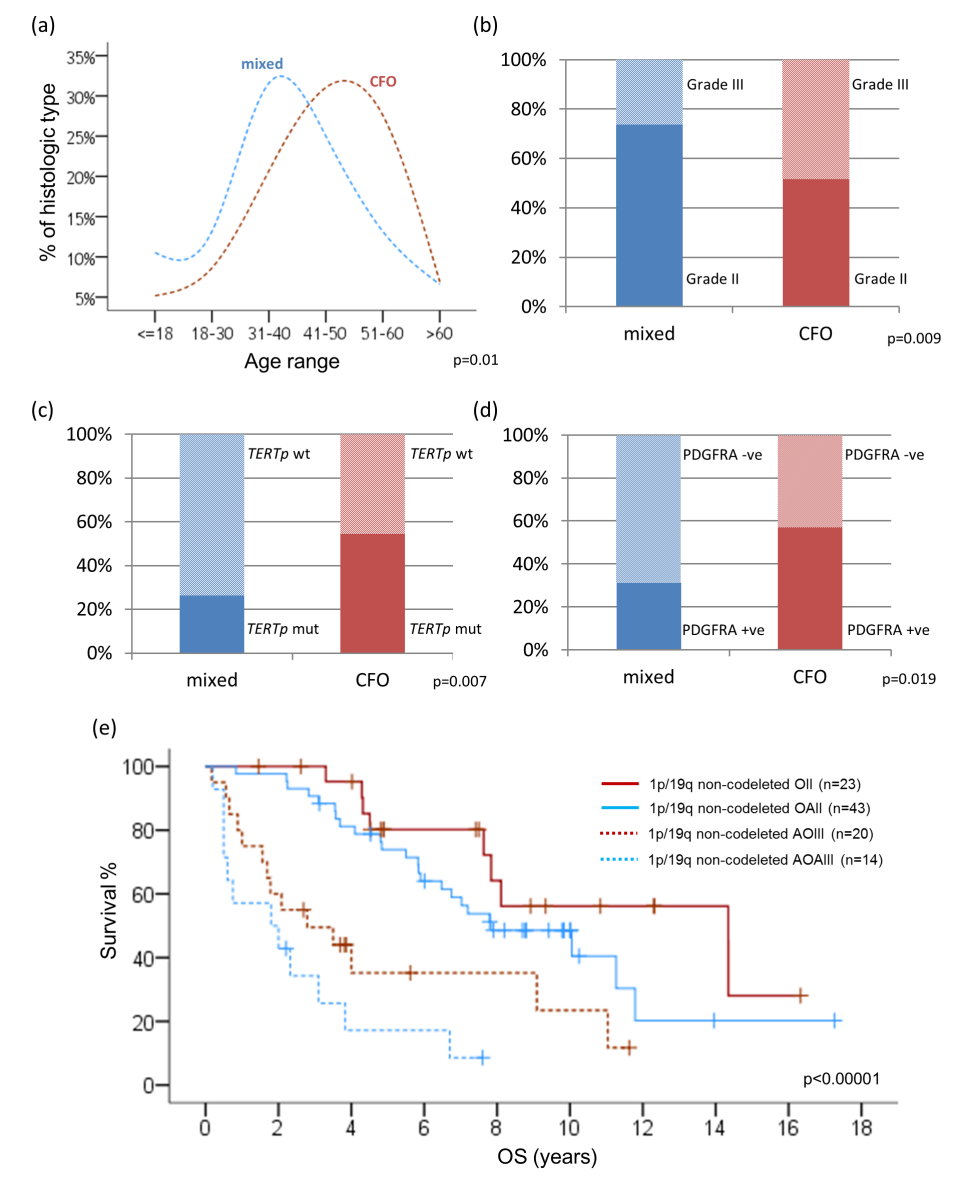
Correlations between clinicopathological factors and molecular variables among 1p/19q intact oligodendroglial tumors Patients with classic oligodendroglial histology were significantly older than those with mixed oligoastrocytic histology (*p* = 0.01) **a.** Grade III histology was more common in oligodendrogliomas (48.3%) than oligoastrocytomas (26.3%) among 1p/19q non-codeleted tumors **b.** Classic oligodendroglial histology in a background of non-codeleted 1p/19q was associated with *p* mutation (*p* = 0.007) **c.** and positive PDGFRA expression (*p* = 0.019) **d.** Kaplan-Meier curves for overall survival (OS) of tumor histology in 1p/19q intact oligodendroglial tumors **e.** CFO, classic for oligodendroglial morphology; mixed, mixed oligoastrocytic histology; mut, mutant; wt, wild-type; -ve, negative; +ve, positive; OS, overall survival.

Among the 134 cases of 1p/19q non-codeleted oligodendroglial tumors, 43.3% (58/134) of cases demonstrated classic oligodendroglial histology, including 30 oligodendrogliomas and 28 anaplastic oligodendrogliomas. Interestingly, patients with classic oligodendroglial histology were significantly older than those with mixed oligoastrocytic histology (mean age 44.5 years *vs* 38.0 years, *p* = 0.01) (Figure 2a). The difference in age was still observed after excluding 11 paediatric (18 years or below) cases (2 oligodendrogliomas, 1 anaplastic oligodendroglioma and 8 oligoastrocytomas) (46.2 years *vs* 41.4 years, *p* = 0.023) (Supplementary Figure S1a). Prognostically, classic oligodendroglial histology was also associated with better prognosis across the 1p/19q non-codeleted cohort (*p* < 0.000001) (Figure 2e). Correlating with histologic grade, 48.3% (28/58) of 1p/19q non-codeleted oligodendrogliomas showed grade III histology, compared to 26.3% (20/76) in 1p/19q non-codeleted oligoastrocytomas (*p* = 0.009) (Figure 2b). Molecularly, classic oligodendroglial histology in a background of non-codeleted 1p/19q was also associated with *TERTp* mutation (*p* = 0.007) (Figure 2c) and showed frequent *IDH* mutation (67.4%) (Supplementary Figure S1b). In a subset of the 1p/19q non-codeleted tumors (N = 80), PDGFRA immunohistochemical expression was also evaluated and classic oligodendroglial histology was associated with positive PDGFRA expression (*p* = 0.019) (Figure 2d).

### Infrequent astrocytic markers identified in 1p/19q non-codeleted oligodendroglial tumors

**Figure 3:**
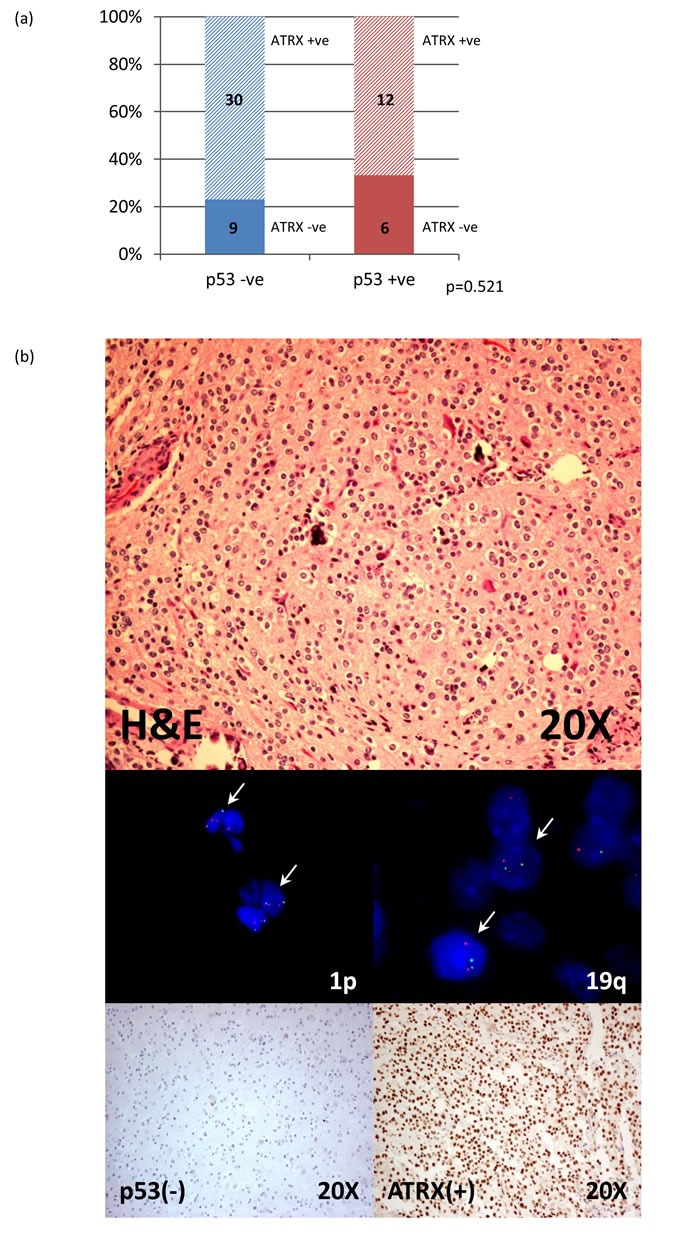
Co-evaluation of p53 and ATRX immunohistochemistry in 1p/19q non-codeleted oligodendroglial tumors 52.6% (30/57) of 1p/19q non-codeleted oligodendroglial tumors lacking p53 positivity and loss of ATRX expression **a.** Photos of 1p/19q intact oligodendroglioma with negative p53 expression and positive ATRX staining **b.** -ve, negative; +ve, positive.

To further interrogate lineage of the oligodendrogliomas lacking 1p/19q codeletion, p53 and ATRX immunohistochemical expression were also evaluated. p53 immunohistochemistry positivity was detectable in only 30% of 1p/19q non-codeleted oligodendroglial tumors among 90 cases examined, including seven anaplastic oligodendrogliomas, 13 oligoastrocytomas and seven anaplastic oligoastrocytomas (Supplementary Figure S1c). Among 70 cases of 1p/19q non-codeleted gliomas examined for ATRX expression, internal positivity was detected in 61 cases and only 26.2% (16/61) of cases demonstrated ATRX** loss. 83.3% (15/18) of oligodendrogliomas and 69.8% (30/43) of oligoastrocytomas showed** ATRX positivity even in a background of intact 1p/19q (Supplementary Figure S1d). Co-evaluation of p53 and ATRX immunohistochemistry identified 52.6% (30/57) of 1p/19q non-codeleted oligodendroglial tumors lacking the important astrocytic markers (Figure 3).

### 1p/19q non-codeleted oligodendroglial tumors with wild-type *TP53* are frequently *IDH/TERTp* mutated

**Figure 4:**
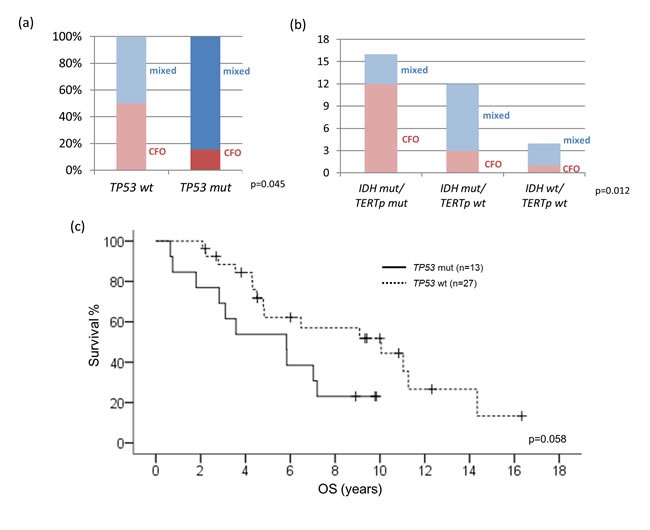
Correlations between *TP53* mutational status and clinicopathological and molecular variables Half of *TP53* wild-type tumors showed classic oligodendroglial histology (*p* = 0.045) **a.** Mutations of *IDH* and *TERTp* co-occurred in 75% of oligodendrogliomas without 1p/19q codeletion and *TP53* mutation (*p* = 0.012) **b.** Patients with Wild-type *TP53* tend to have better survival (*p* = 0.058) **c.** CFO, classic for oligodendroglial morphology; mixed, mixed oligoastrocytic histology; mut, mutant; wt, wild-type; OS, overall survival.

We additionally conducted mutational analysis of *TP53* (exon 4 to 9) in 45 cases of oligodendroglial tumors lacking 1p/19q codeletion. *TP53* mutations detected were summarized in Supplementary Table S2. Wild-type *TP53* was observed in 71.1% (32/45) of 1p/19q non-codeleted oligodendroglial tumors and trended to better survival (*p* = 0.058) (Figure 4c). Importantly, 50% (16/32) of *TP53* wild-type tumors showed classic oligodendroglial histology (*p* = 0.045) (Figure 4a). Among this subset of 1p/19q non-codeleted, *TP53* wild-type oligodendroglial tumors, *IDH* and *TERTp* mutations were present in 87.5% (28/32) and 50% (16/32) of cases, respectively. The mutations co-occurred in 75% (12/16) of oligodendrogliomas without 1p/19q codeletion and *TP53* mutation (*p* = 0.012) (Figure 4b).

## DISCUSSION

In the present study, we examined a large cohort of oligodendroglial tumors (N = 337) and identified 60.2% of cases harbored 1p/19q codeletion. Since first reported by Reifenberger and colleagues in 1994 [16], combined chromosomal whole arm deletion in 1p and 19q has been referred as genetic hallmark of oligodendrogliomas [17]. Mediated by an unbalanced translocation between chromosome 1 and 19, with formation of derivative chromosomes der(1;19)(q10;p10) and der(1;19)(p10;q10) and the latter one being subsequently deleted during gliomagenesis [13, 18], 1p/19q codeletion was one of the most representative and remarkable molecular markers in diffuse gliomas with diagnostic, prognostic and predictive utilities in clinical practice [7, 19]. The marker served as a stratifier in clinical trials and was shown to predict therapeutic response to PCV chemotherapy (procarbazine, lomustine and vincristine) and radiotherapy in oligodendroglial tumors [1, 5]. 1p/19q codeletion will be one of the molecular markers being incorporated into the integrated morphological and molecular diagnosis of CNS tumors as proposed by the International Society of Neuropathology-Haarlem Consensus Guidelines [20].

While classical oligodendrogliomas would be defined by 1p/19q codeletion [7], there were clearly oligodendroglial tumors lacking the molecular hallmark in various retrospective cohorts and important clinical trials, some of which with histology having been vigorously interrogated by a central review panel of experienced neuropathologists [1, 5, 6, 10, 11, 14, 15, 21-37] (Table 4). In the EORTC 26951 study, 75% of 316 oligodendroglial tumors had non-codeleted 1p/19q and 175 cases were oligodendroglioma [5]. In the RTOG 9402 trial, 29% of 150 oligodendroglial tumors had intact 1p or 19q alleles [1], when the histology of the same cohort was subject to central panel review, 20% (19/97) of the CFO (classic for oligodendroglial morphology) tumors had intact 1p/19q [38]. So clearly, “oligodendroglial tumors” without 1p/19q codeletion are a distinct, sizeable group which cannot be put aside. In our study, we identified 24% (30/125) of oligodendrogliomas and 38.9% (28/72) of anaplastic oligodendrogliomas had intact 1p or 19q, yielding an overall frequency of 1p/19q non-codeleted oligodendrogliomas of 29.4% (58/197). Our results corroborated the observations of the existence of 1p/19q intact oligodendrogliomas from previous studies and suggested the need of additional biomarker(s) to characterize this subset of oligodendrogliomas.

**Table 4:** Review of various retrospective cohorts and important clinical trials

Study	No. of O examined	No. of AO examined	No. of non-1p/19q codeleted O identified	No. of non-1p/19q codeleted AO identified	Method used
Ducray F and Sanson M et al. 2008 [25]	22	24	9 (40.9%)	12 (50%)	array-CGH
Durand KS and Labrousse FJ et al. 2010 [30]	2	5	0	0	LOH
Labussiere M and Sanson M et al. 2010 [27]	90	119	43 (47.8%)	55 (46.2%)	array-CGH
Buckley PG and Farrell MA et al. 2011 [33]	15	12	3 (20%)	3 (25%)	FISH and array-CGH
Ducray F and Sanson M et al. 2011 [35]	73	133	30 (51%)	42 (45%)	array-CGH
Eigenbrod S and Kretzschmar HA et al. 2011 [37]	10	10	1 (10%)	4 (40%)	LOH
Goze C and Duffau H et al. 2012 [29]	21	-	6 (29%)	-	LOH
Jiao Y and Yan H et al. 2012 [36]	21	29	8 (38.1%)	3 (10.3%)	LOH
Li S and Jiang T et al. 2012 [14]	-	36	-	22 (61.1%)	DHPLC
Sahm F and Hartmann C et al. 2012 [34]	9	9	2 (22.2%)	1 (11.1%)	LOH and MLPA
Arita H and Ichimura K et al. 2013 [10]	34	31	7 (23%)	7 (27%)	MLPA
Cairncross G and Mehta M et al. 2013 [1]	-	150	-	43 (29%)	FISH
Frenel JS and Campone M et al. 2013 [32]	0	43	0	17 (42.5%)	FISH
Jiang H and Lin S et al. 2013 [28]	-	24	-	7 (29.2%)	FISH
Mur P and Melendez B et al. 2013 [31]	19	14	14 (42.4%)	FISH
van den Bent MJ and Hoang-Xuan K et al.2013 [5]	-	316 (AO and AOA)	-	236 (75%, AO and AOA)	FISH
Chan AK and Ng HK et al.2014 [15]	19	11	7 (36.8%)	4 (36.4%)	FISH
Gillet E and Idbaih A et al. 2014 [23]	32	-	16 (50%)	-	LOH
Sahm F and von Deimling A et al. 2014 [11]	43 (OA)	11 (25.6%, OA)	FISH
Brat DJ and Zhang J et al. 2015 [51]	65	44	27 (41.5%)	13 (29.5%)	Genomic Analysis
Chan AK and Ng HK et al.2015 [6]	18	3	7 (41.2%)	1 (33.3%)	FISH
Gleize V and Sanson M et al. 2015 [24]	60	43	4 (6.7%)	9 (20.9%)	LOH
Weller M and Reifenberger G et al. 2015 [26]	3	3	0	1 (33.3%)	array-CGH
Dubbink HJ and van den Bent MJ et al. 2016 [22]	-	93	-	40 (47.1%)	LOH, FISH and NGS

O, oligodendroglioma; AO, anaplastic oligodendroglioma; OA, Oligoastrocytoma; FISH, fluorescence in situ hybridization; LOH, loss of heterozygosity; MLPA, multiplex ligation-dependent probe amplification; DHPLC, denaturing high-performance liquid chromatography; NGS, next-generation sequencing; array-CGH, array-based comparative genomic hybridization

Genomic landscape of grades II and III diffuse gliomas was comprehensively interrogated in the landmark study reported by the TCGA Research Network using multi-omic platforms [39]. The genomewide analyses identified concordant classification of three prognostically relevant molecular subgroups of lower grade gliomas based on unsupervised clustering of multi-omic data, which can be robustly identified by *IDH* mutation and 1p/19q codeletion. Importantly, *IDH* mutated, 1p/19q codeleted gliomas were characterized by mutations in *CIC, FUBP1, NOTCH1,* and *TERT* promoter, suggesting these mutations could serve as oligodendroglial lineage markers. *IDH* mutated gliomas lacking 1p/19q codeletion showed high frequency of *TP53* mutation (94%) and *ATRX* inactivation (86%), compatible with their role as astrocytic markers. These substantial genomic data provided the basis of our choice of astrocytic and oligodendroglial markers in our current study (Supplementary Table S3). It was worthwhile to note that in the TCGA report, OncoSign analysis based on recurrent copy-number variations, mutations, and gene fusions, was performed and the group identified four dominant OncoSign classes, namely OSC1 to OSC4, which were correlated with the *IDH*-1p/19q defined subgroups. 1p/19q non-codeleted oligodendrogliomas were enriched in OSC3 and were associated with *IDH* mutation and *TERT*p mutation, validating the observations in our study. Another remarkable study from Weller *et al*. interrogated grades II and III diffuse gliomas using genome- and transcriptome-wide profiling and reported molecular subgroups harboring distinct genomic aberrations and expression patterns which provided prognostically significant information on top of *IDH* mutation and 1p/19q codeletion [26]. Genomic profiling revealed five molecular subgroups with Group I tightly correlating with *IDH* mutation and 1p/19q codeletion and exhibiting the best clinical outcome, and Group V harboring glioblastoma-like copy number changes including 7q gain and 10q loss, and showing the poorest survival. Importantly, the genomic profiling provided clinically relevant information with biological implication for prognostication of diffuse gliomas beyond histologic classification and grading.

1p/19q non-codeleted oligodendrogliomas in our study were associated with *TERTp* mutation (*p* = 0.007) and demonstrated frequent *IDH* mutation (67.4%). Co-mutations of *IDH* and *TERTp* occurred in 75% of 1p/19q intact, *TP53* wild-type oligodendrogliomas in our cohort. Notably, *IDH* mutation was also frequently detected in the 1p/19q intact oligodendroglial tumors and conferred chemo-radiation sensitivity in the absence of 1p/19q codeletion as shown by Cairncross and colleagues [21]. In terms of classification and diagnostic utility, presence of *IDH* mutation, even without 1p/19q codeletion, has been suggested to indicate the diagnosis of oligodendrogliomas over other tumors with similar histo-morphological features [40]. Multiple studies have shown the association between *TERTp* mutation and 1p/19q codeletion as well as classic oligodendroglial morphology [6, 9, 10, 41, 42]. Our current study validated previous observations and additionally demonstrated the association between *TERTp* mutation and classic oligodendroglial morphology in a molecular background of intact 1p/19q. Notably, in the study by Killela and colleagues examining more than 470 diffuse gliomas, co-mutations of *IDH* and *TERTp* were identified in 79.3% (69/87) of oligodendrogliomas while 1p/19q codeletion was only observed in 54% (47/87) of the cases [41]. Our study and previous observations suggested the potential of *IDH* mutation plus *TERTp* mutation in assisting the diagnosis of oligodendroglioma for a tumor with clear cell morphology but lacking 1p/19q codeletion.

It was crucial to avoid misclassification of true 1p/19q codeleted oligodendrogliomas as non-deleted tumors (ie false negativity) in our study. We employed FISH and used commercial probe targeting the “minimally deleted regions” in order to maximize the sensitivity in detecting 1p/19q codeletion [2]. The technique had been well standardized and adopted in various studiesand could be applied robustly on formalin-fixed paraffin-embedded tissues in routine diagnostic neuropathology practice. Importantly, FISH probes targeting at the same loci were also adopted by the EORTC and RTOG clinical trials [1, 5]. Since 1p/19q loss classically involved deletion of the entire 1p and 19q arms, tumors with retained minimally deleted regions in our study were extremely unlikely to be false negative cases.

We further examined astrocytic markers, p53 expression and ATRX loss, in the 1p/19q non-codeleted oligodendroglial tumors. Co-evaluation of the two astrocytic immuno-markers revealed more than 50% of the 1p/19q non-codeleted oligodendroglial tumors did not show p53 positivity and ATRX loss, i.e. they were not molecularly astrocytic. Among 38 cases of mixed oligoastrocytomas with analyzable data of ATRX expression and *TERTp* mutation, the two biomarkers showed mutual exclusivity in all but one case [Supplementary Figure S1e]. *TERTp* and *ATRX* mutations represented independent genetic mechanisms in maintaining telomere lengths in tumorigenesis of diffuse gliomas [42]. In the study by Sahm and colleagues investigating 43 mixed oligoastrocytomas, 1p/19q codeletion and ATRX loss were mutually exclusive in all but one case, suggesting parting with the diagnosis of oligoastrocytoma. According to these authors, all non-codeleted oligodendroglial tumors are in fact astrocytic [11]. With the morphological and molecular evidence of several cases of “true” oligoastrocytoma being reported [46-48], complete farewell to oligoastrocytoma can be argued. Notably, in our series, we also did not find any true cases of “oligoastrocytoma”.

**Figure 5:**
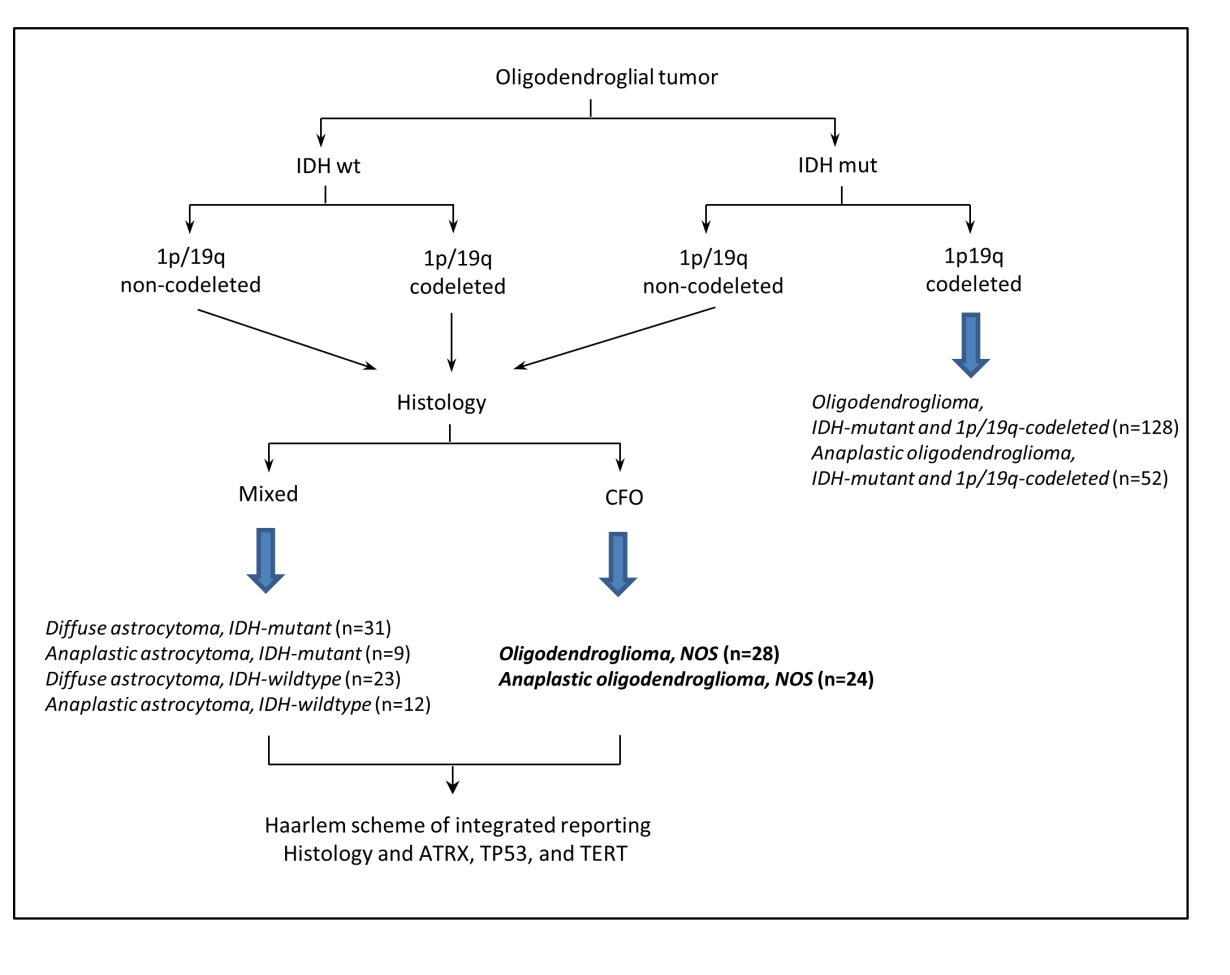
Overview of the sequential work-flow based on The 2016 WHO classification of CNS tumors and Haarlem scheme [20, 56] mixed, mixed oligoastrocytic histology; CFO, classic for oligodendroglial morphology.

Clinical trials by Cairncross *et al.* and van den Bent *et al*. have set the standard therapeutic protocol for patients with 1p/19q codeleted anaplastic oligodendroglial tumors [1, 5]. The former research group has additionally demonstrated that the survival benefit of PCV (procarbazine, lomustine, and vincristine) in 1p/19q non-codeleted anaplastic oligodendroglial tumors was associated with *IDH* mutation [21]. Clinical benefits of radiation plus PCV have been extended to treatment of low grade diffuse gliomas [49]. The chemoradiotherapy was associated with prolonged overall survival and progression-free survival in subsets of oligodendroglioma, oligoastrocytoma, and *IDH* mutated low grade gliomas. Our group has previously reported the potential clinical value of *TERT*p mutation in predicting differential responses to genotoxic therapies in grades II and III diffuse gliomas, on top on *IDH* status [50]. Our current study identified a major portion of oligodendrogliomas lacking 1p/19q codeletion was characterized by *IDH* and *TERT*p co-mutation, with a background of ATRX nuclear positivity wild-type *TP53* (Figure 5), indicating that these patients could potentially benefit from chemoradiotherapy.

In summary, this study examined a large cohort of oligodendroglial tumors and demonstrated that astrocytic tumors could not account for all 1p/19q non-codeleted oligodendroglial tumors and oligodendroglioma lacking 1p/19q codeletion may form a distinct subgroup of diffuse glioma. Co-evaluation of *IDH* and *TERTp* mutation had diagnostic value for oligodendroglioma and could serve as an adjunct in oligodendroglial tumors after evaluation of the 1p/19q status.

## MATERIALS AND METHODS

### Patients and tissue samples

Oligodendroglial tumors diagnosed from our service archives at the Prince of Wales Hospital (The Chinese University of Hong Kong) and Huashan Hospital (Fudan University, Shanghai) were included in the present study. Totally 337 oligodendroglial tumors were included in our study and 71 of them were from Hong Kong and 266 from Shanghai. Only cases which were reviewed by two neuropathologists (HK N and H C) as oligodendroglial were included based on The 2007 WHO classification of tumors of the central nervous system [51]. The present study used only formalin-fixed, paraffin-embedded material. Clinical and survival data of the patients were retrieved from the respective institutional medical record systems. This study was approved by the Ethics Committee of Shanghai Huashan Hospital and the New Territories East Cluster-Chinese University of Hong Kong Ethics Committee. The cohort in this study partially overlapped with previous studies [6, 15, 52].

### Fluorescence *In Situ* hybridization for chromosome 1p and 19q

Fluorescent *in situ* hybridization (FISH) was performed on formalin-fixed, paraffin-embedded tumor tissue to detect deletion of chromosome 1p and 19q [15]. 4-μm-thick sections were deparaffinized, treated with sodium thiocyanate and followed by digestion with pepsin solution at 37°C. Dual-color-probe hybridization was then performed using Vysis 1p36/1q25 and 19q13/19p13 FISH Probe Kit (Abbott Molecular) and the spectrum-green-probe was labeled on chromosome 1q and 19p, respectively. Both probes and target tumor DNA were denatured in an 80°C oven for 30 minutes and followed by an overnight incubation at 37°C. Nuclei were counterstained with Vectashield mounting medium containing 4′, 6-diamidino-2- phenylindole (Vector Laboratories, Burlingame, CA, USA) and the number of FISH signals was assessed under a Zeiss Axioplan fluorescence microscope (Carl Zeiss Microscopy LLC, NY, USA) equipped with a triple-pass filter (DAPI/Green/Orange). Hybridizing signals of at least 100 non-overlapping nuclei were enumerated and a sample will be considered as 1p or 19q deleted when more than 25% of counted nuclei presented one target (orange) signal and two reference (green) signals [2].

### Mutation analysis of *IDH1/2*, *TERT* promoter and *TP53*

Mutation status of *IDH1/2, TERT* promoter and *TP53* were studied with direct sequencing [6, 52, 53]. Tissues from representative areas with tumor content > 70% were collected from 5 to 7 dewaxed formalin-fixed, paraffin-embedded sections and re-suspended in 10mM Tris-HCl buffer (pH 8.5) which also contained proteinase K with a final concentration of 2 mg/ml. After that, the mixture was incubated at 55°C for 2-18 h and then at 98°C for 10 min. After the above incubation, the buffer comprising the cell lysate was used for polymerase chain reaction (PCR) analysis of *IDH1, IDH2, TP53* and *TERTp*. We performed direct sequencing for *IDH1*, *IDH2*, *TERT* promoter region and exon 4-9 of *TP53* as per previous protocols [6, 15, 52-54]. All mutational status was validated by sequencing of a newly amplified DNA fragment. Sequences of primers used for PCR analysis of *IDH1/2*, *TERT* promoter and *TP53* were listed in Supplementary Table S1. For sequencing of *IDH1/2* and *TP53*, target DNA fragments were amplified in a total 10μl reaction volume which containing 1μl of cell lysate, 1μl of MgCl2 (25mM), 1μl of 10*PCR buffer II, 0.2μl of each deoxyribonucleoside triphosphate (10mM), 0.3μl of each forward and reverse primers of target DNA fragment (10nmol), 0.075μl of AmpliTaq Gold DNA polymerase (Life Technologies Corporation, Hong Kong, China). PCR for *TERT* promoter region was performed in 10μl reaction mixture containing 1μl of cell lysate, 0.3μl of each forward and reverse primers of target DNA fragment (10nmol), and 5μl of KAPA HiFi HotStart ReadyMix DNA Polymerase (Kapa Biosystems Wilmington, DE, USA). PCR reaction started with a denature procedure of 95°C for 10 min, then followed by 45 cycles of 95°C for 20 sec, annealing temperature (Supplementary Table 1) for 20 sec and 72°C for 30 sec, and a final extension step of 72°C for 3 min. Products were then treated with Exonuclease I (TakaRa Biotechnology Limited, Dalian, China) of 2μl (0.25U/μl) per 5μl PCR product at 37°C for 15min followed by 80°C for 15min. Sequencing of target DNA fragment was performed using BigDye Terminator Cycle Sequencing kit v.1.1 (Life Technologies). The Genetic Analyzer 3130xl was used for the following sequencing and the results were analyzed by Sequencing Analysis Software. Hotspot mutations of *IDH1/2* and *TERTp*, all missense mutations of *TP53* could be detected. All detected missense mutations of *TP53* were listed in Supplementary Table S2.

### Immunohistochemistry of ATRX, p53 and PDGFRA

Immunohistochemistry was performed for detecting the expression of p53, PDGFRA, and ATRX. 4μm thick formalin-fixed, paraffin-embedded (FFPE) sections of each sample were de-waxed by xylene and rehydrated in graded alcohols. Sections were then treated with citrate buffer (pH 6.0) by heating for antigen retrieval. After antigen retrieval, they were subjected to immuno-staining by BenchMark XT automated tissue staining systems (Ventana Medical Systems, Tucson, AZ, USA) using validated protocols for detecting the expression of p53 and ATRX. Sections were incubated with antibodies of anti-p53 (Dako DO-7, 1:100) and anti-ATRX (SIGMA CAT: HPA001906, 1:400) at 37°C for 32 min and followed by an incubation with UltraView HRP-conjugated multimer antibody reagent (Ventana Medical Systems). Subsequent antigen detection was performed using UltraView diamino benzidine chromogen step (Ventana Medical Systems). Expression of PDGFRA was detected using the automated Bond-max system (Leica Bond-Max) with anti-PDGFRA antibody (Santa Cruz, C-20, 1:200) and subsequent antigen detection was performed using Ultra View diamino benzidine chromogen step (Bond-Max). At last, slides were counterstained with Mayer's hematoxylin. A tumor was scored p53 positive if > 10% of tumor cells showed strong nuclear staining [53]. Samples with more than 10% of tumor cells showing positive nuclear staining of ATRX were scored as positive. Endothelial cells, cortical neurons and infiltrating inflammatory cells were generally positive and served as internal positive controls [12]. Cytoplasmic and membrane staining were considered for evaluation of PDGFRA expression. Both the distribution and intensity of staining were semi-quantitatively scored as previously reported [55].

### Statistical analysis

Statistical analysis was performed using IBM SPSS Statistics Version 20 (IBM Corporation, NY, USA). Univariate analysis was conducted using Chi-square or Fisher's test to compare categorical variables. Independent-Samples T Test was used to compare mean age between 2 populations. Kaplan-Meier estimator and log rank test were performed for univariate survival analysis. Whereas a Cox proportional hazards model was employed for multivariate survival analysis. Statistical significance was considered when *p* < 0.05 (two-side).

## SUPPLEMENTARY MATERIAL FIGURES AND TABLES


