# Supplementary material for: Not all 1p/19q non-codeleted oligodendroglial tumors are astrocytic
Source: Oncotarget. 2016 Aug 18;7(40):64615–30. doi: 10.18632/oncotarget.11378 (PMC5323103; doi:10.18632/oncotarget.11378)
Supplement: Supplementary file 1 [file oncotarget-07-64615-s001.pdf]

Not all 1p/19q non-codeleted oligodendroglial tumors are astrocytic

Supplementary Material

Supplementary Table S1. Primers used for Direct Sequencing

Gene	Primer (F/R)	Sequence (5' to 3')	Primer length (bp)	PCR product size (bp)	Annealing Temperature
IDH 1	F	CGGTCTTCAGAGAAGCCATT	20	122	60°C
	R	CACATTATTGCCAACATGAC	20		
IDH 2	F	AGCCCATCATCTGCAAAAAC	20	150	60°C
	R	CTAGGCGAGGAGCTCCAGT	19		
TP53	F5	CCTGGTCCTCTGACTGCTCT	20	242	64°C
EXON 4	R5	TTCTGGGAAGGGACAGAAGA	20		
TP53	F3	GTTCTTTCGTCGGCTCTTC	19	357	60°C
EXON 5	R3	GGGCCAGACCTAAGAGCAAT	20		
TP53	F3	GCTGGGGCTGGAGAGACGACAG	22	260	64°C
EXON 6	R3	TACTGCTCACCTGGAGGGCCACTG	24		
TP53	F7	CAAGGCGCACTGGCCTCAT	20	216	64°C
EXON 7	R1	GTCAGAGGCAAGCAGAGGCT	20		
TP53	F3	CAAGGGTGGTTGGGAGTAGA	20	327	60°C
EXON 8	R3	AGGAAAGAGGCAAGGAAAGG	20		
TP53	F2	GACCAAGGGTGCAGTTATGC	20	196	60°C
EXON 9	R2	CGGCATTTTGAGTGTTAGACTG	22		
TERT	F2	GTCCTGCCCCTTCACCTTC	19	274	68°C
Promoter	R2	AGCACCTCGCGGTAGTGG	18		

F, Forward; R, Reverse

Supplementary Table S2. Somatic mutations of *TP53* in 1p/19q non-codeleted oligodendroglial tumors

Case NO.	Histology	Mutations			Exon
		Nucleotide change	Protein change	Mutation type	
OA14	OA(III)	c.275C>T	p.Pro92Leu	missense	4
OA116	OA(II)	c.524G>A	p.Arg175His	missense	5
OA7	OA(II)	c.431A>G	p.Gln144Arg	missense	5
OA13	AOA(III)	c.431A>G	p.Gln144Arg	missense	5
O161	O(III)	c.659A>G	p.Tyr220Cys	missense	6
OA118	OA(II)	c.579T>A<br>c.580C>T	p.His193Gln<br>p.Leu194Phe	missense	6
OA4	OA(II)	c.659A>G	p.Tyr220Cys	missense	6
OA89	OA(II)	c.578A>C	p.His193Pro	missense	6
O101	O(II)	c.580C>T	p.Leu194Phe	missense	6
OA24	OA(II)	c.743G>A	p.Arg248Gln	missense	7
OA15	OA(II)	c.743G>A	p.Arg248Gln	missense	7
OA20	AOA(III)	c.817C>T	p.Arg273Cys	missense	8
OA122	OA(II)	c.799C>T	p.Arg267Trp	missense	8

O,oligodendroglioma; OA, oligoastrocytoma

Supplementary Table S3. Molecular markers used for differentiation of gliogenesis lineage in this study

Technique	Astrocytic lineage	oligodendroglial lineage
Immunohistochemistry	p53, ATRX	PDGFRA
Direct sequencing	*TP53*	*TERT*
FISH		1p/19q

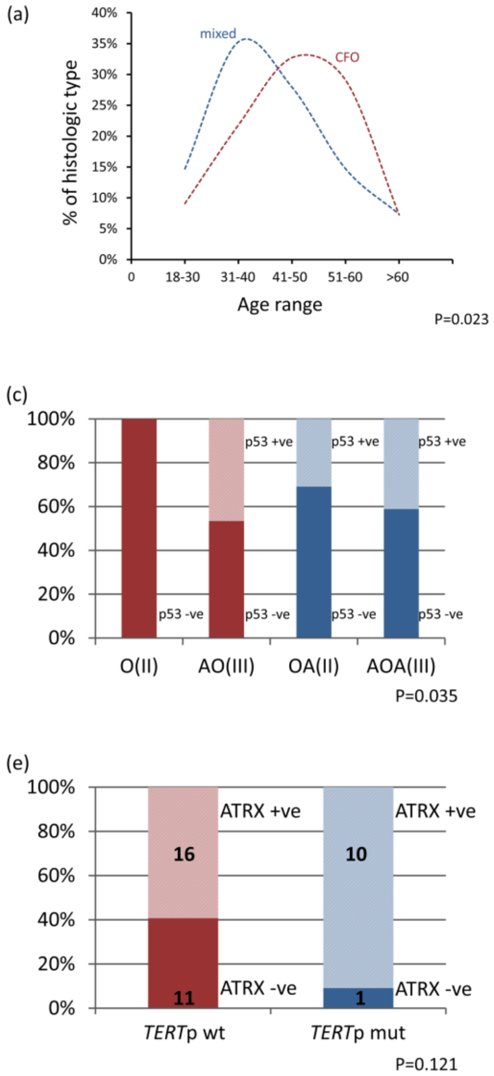

Supplementary Figure S1. Correlation between clinicopathological and molecular variables of 1p/19q intact oligodendroglial tumors. Patients with classic oligodendroglial histology were significantly older than those with mixed oligoastrocytic histology when excluding paediatric cases (p=0.023) **a.** 1p/19q intact oligodendroglial tumors with classic oligodendroglial histology showed frequent *IDH* mutation (67.4%) **b.** Distribution of p53 positivity **c.** and ARTX positivity **d.** in 1p/19q non-codeleted oligodendroglial tumors according to histology. ATRX expression and *TERTp* mutation were mutually exclusive in all but one case among 1p/19q non-codeleted oligoastrocytomas **e.**
